# A Simple Technique for Intraoperative Scalp Skin Graft Depilation Using Dermabond®

**DOI:** 10.7759/cureus.9786

**Published:** 2020-08-16

**Authors:** Jude L Opoku-Agyeman, Kayla Humenansky, Brooke Burkey

**Affiliations:** 1 Plastic Surgery, Philadelphia College of Osteopathic Medicine, Philadelphia, USA; 2 Plastic and Reconstructive Surgery, Philadelphia College of Osteopathic Medicine, Philadelphia, USA; 3 Plastic and Reconstructive Surgery, St. Christopher Hospital for Children, Philadelphia, USA

**Keywords:** graft depilation, scalp graft, skin graft, dermabond, surgical glue

## Abstract

Skin grafting is an essential aspect of burn and wound reconstruction. Split-thickness skin grafts (STSGs) harvested from the scalp are used for wound and burn reconstruction. Skin grafts from the scalp bear hair and hair particles. Residual hair fragments and pieces of hair in the graft have been associated with many complications, including foreign body reaction similar to pseudofolliculitis and chronic inflammation that can lead to infections. It is important to remove the hair and the hair particles from the scalp graft before its application to the donor site. Traditionally, surgeons have employed some techniques including saline agitation and mechanical removal of the hair particles with forceps. These techniques are time consuming and can subject the graft to mechanical damage. There is another technique that has been described using an adhesive tape. This technique uses Ioban™ (3M Healthcare, St. Paul, MN), followed by a saline wash to remove hair from grafts prior to grafting. In this paper, we introduce a novel technique for intraoperative hair depilation prior to graft application to recipient site. We used Dermabond® (Ethicon, Bridgewater, NJ) to remove residual hair particles from the STSG donor. Our technique has several advantages: it is expeditious, it allows minimal mechanical damage to the graft, and can be used for patients with allergies to Ioban. Intraoperative Dermabond depilation of scalp STSGs is safe, easy, and effective.

## Introduction

The scalp is one of the many sites for obtaining split-thickness skin grafts (STSGs) for burn reconstruction and wound reconstruction. The scalp donor site has many advantages, including rapid wound healing, low risk of complications, and excellent cosmesis [1]. However, the use of the scalp as a donor site for skin grafting is also associated with some potential complications, including alopecia. In addition, the scalp donor has a potential for foreign body reaction after it has been grafted due to its hair-bearing nature. This foreign tissue reaction has been akin to pseudofolliculitis. This is believed to be a result of hair entrapment within the dermis [2-4]. The entrapped hair can also lead to chronic inflammation and subsequent infection. Before scalp skin graft is harvested, the scalp is usually shaved; however, many hair fragments remain in the graft. There are currently limited techniques employed for intraoperative depilation. Some surgeons vigorously wash the graft in saline to remove the residual hair fragments. Others use a combination of saline and gauze to mechanically pull the hair off, while yet, others use small forceps to pull the individual hair fragments out of the graft. These techniques are tedious and may subject the graft to mechanical damage. Recently, the use of Ioban™ (3M Healthcare, St. Paul, MN) adhesive tape and saline washing for scalp graft depilation was described [5]. This technique is less tedious than the previously described methods of graft depilation and also offers the advantage of less mechanical damage to the graft. However, this technique cannot be utilized in patients with allergies to iodine and Ioban. We developed an improved technique for intraoperative depilation of scalp STSGs prior to grafting using Dermabond® (Ethicon, Bridgewater, NJ).

## Technical report

**Figure 1:**
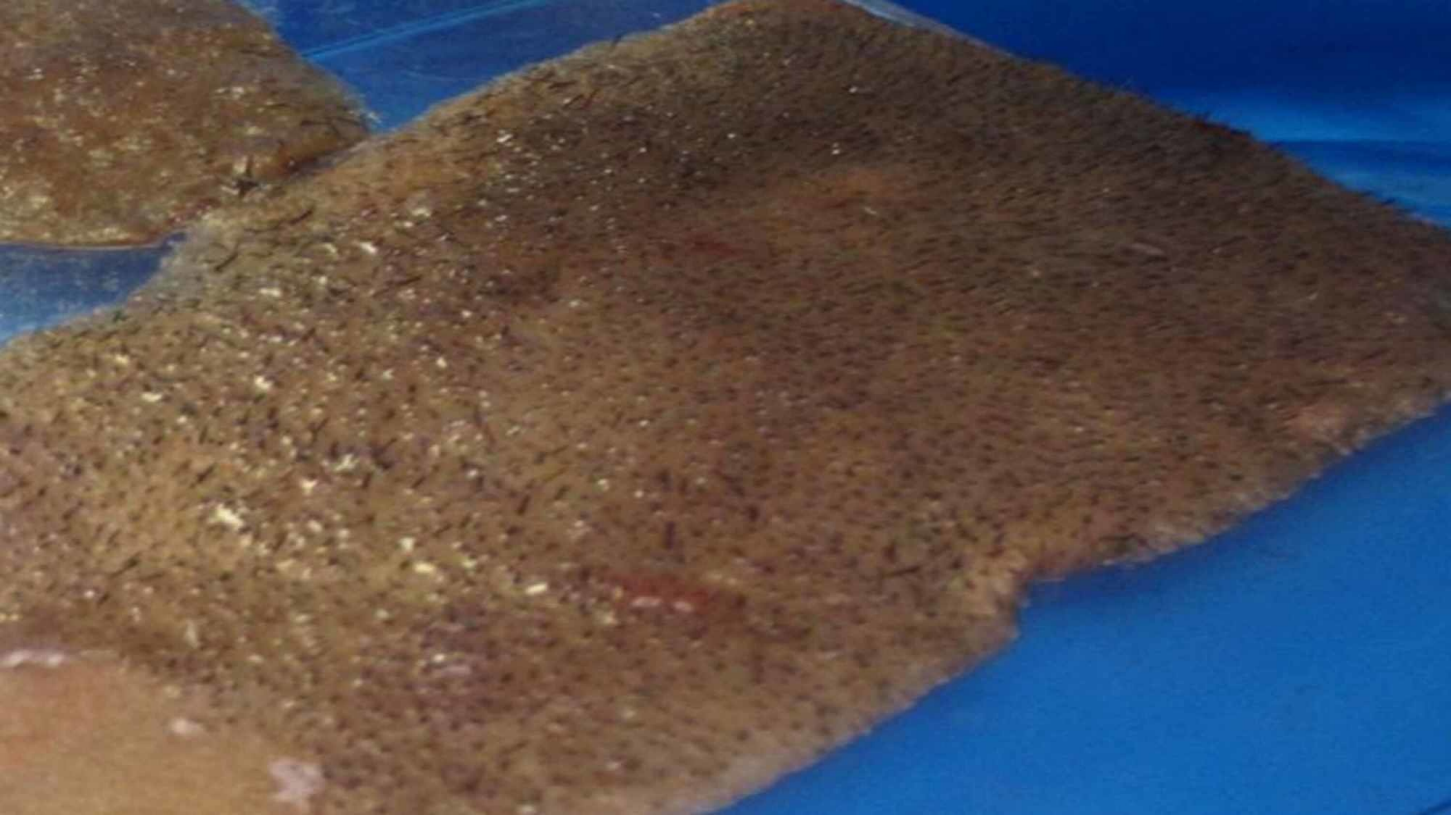
Dried Dermabond on the hair bearing surface of the skin graft

An STSG is harvested from the scalp using a dermatome. The graft is wetted with saline and placed on a flat surface. The graft is then patted dry using a dry gauze. A thin layer of Dermabond is then applied to the epidermal surface of the graft. The Dermabond is allowed to dry and forms a thick coat on the graft (Figure 1). 

**Figure 2:**
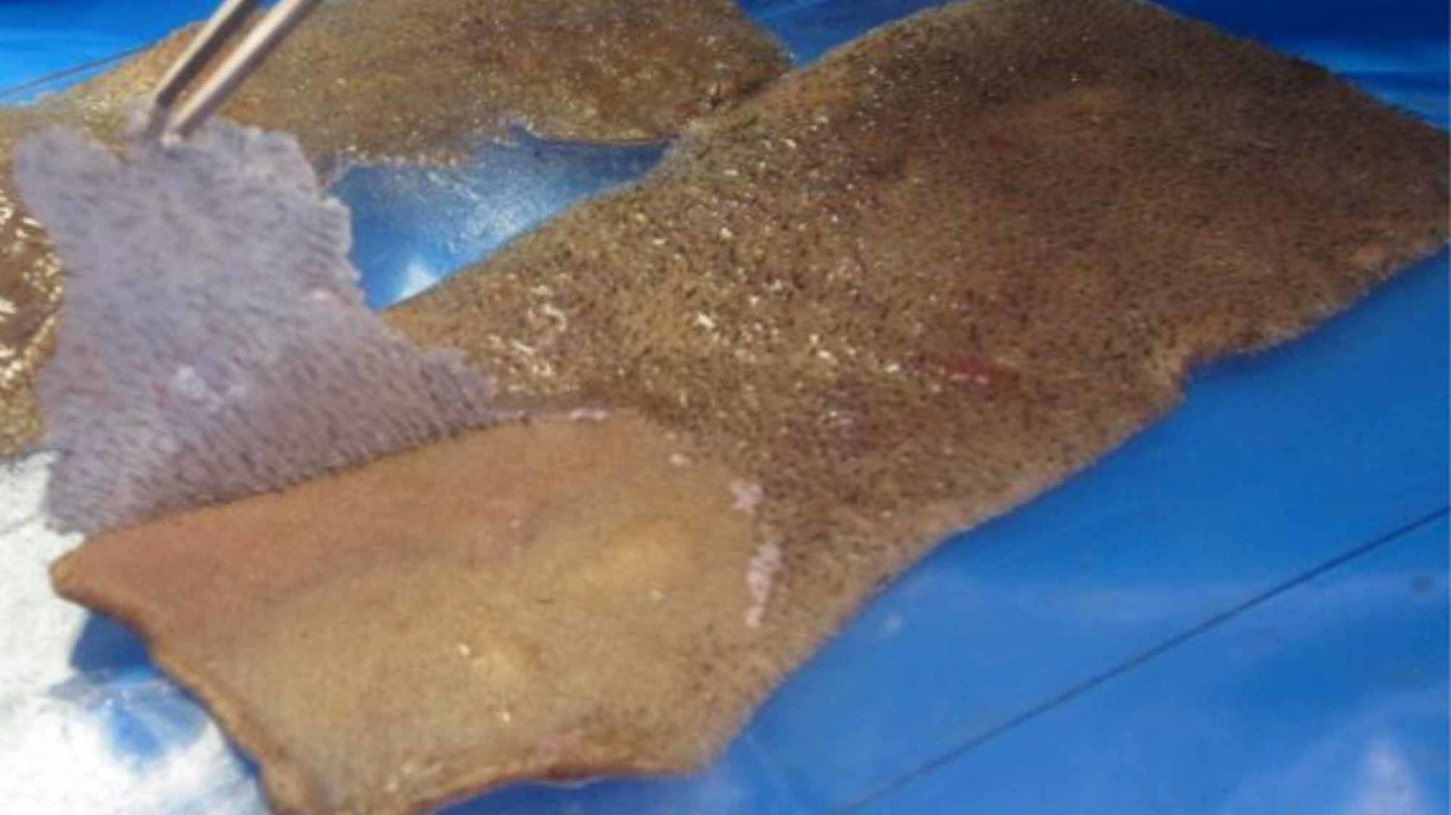
Dermabond being peeled from graft. Notice hair embedded in the Dermabond

It takes approximately three to four minutes for the Dermabond to dry. Beginning at an arbitrary area within the graft, the Dermabond is meticulously peeled off the graft using Adson or Bishop-Harmon forceps. Once an edge is developed, the Dermabond peels off easily. The hair fragments remain firmly embedded within the dried Dermabond, revealing an almost complete hair-free graft (Figure 2).

**Figure 3:**
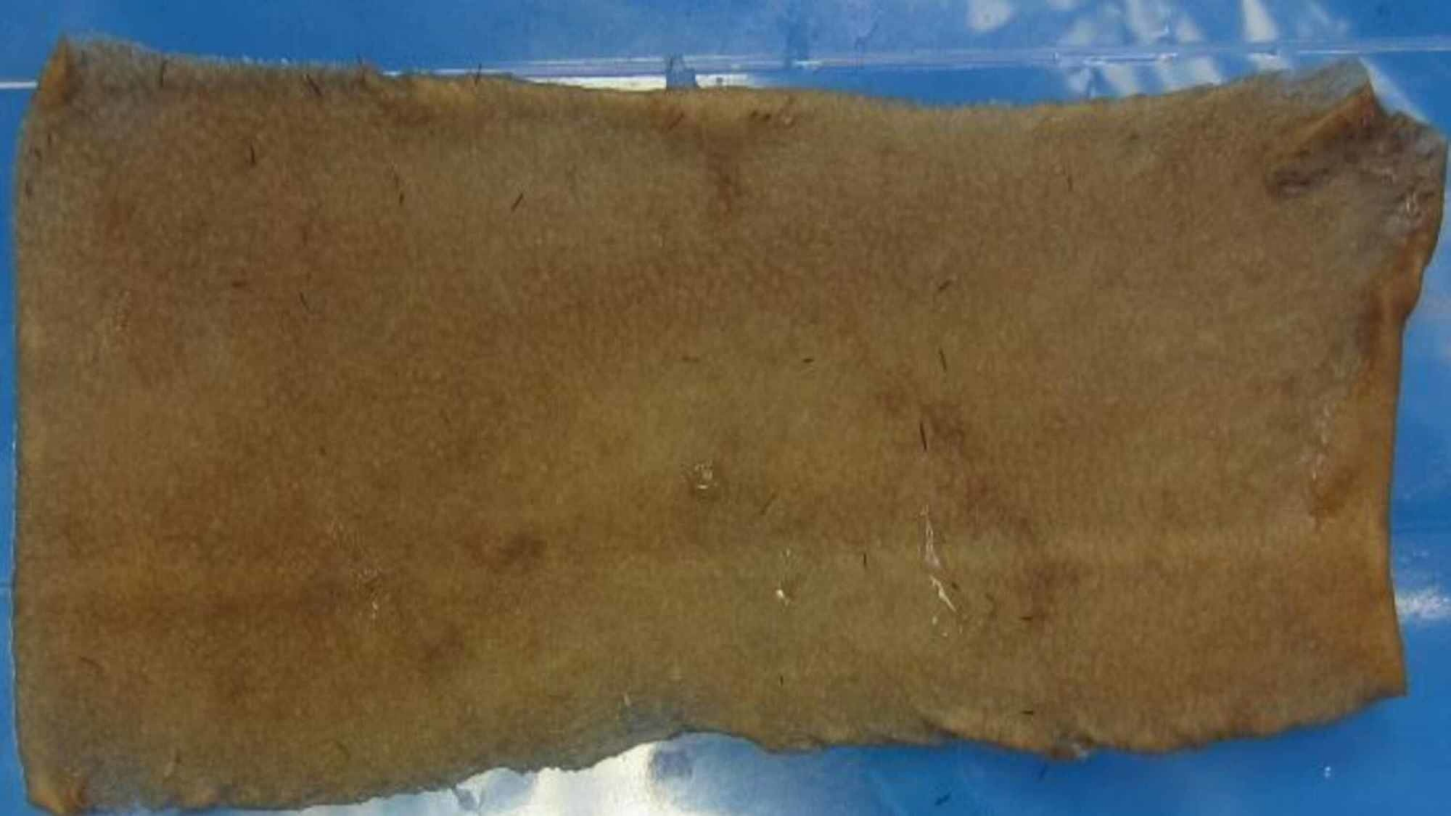
Scalp STSG after Dermabond depilation STSG, split-thickness skin graft

Most of the hair was removed with one application of Dermabond. The few remaining hair fragments were easily removed upon rinsing the graft in saline. No graft sustained any punctures or tears indicative of mechanical injury. Each graft was rinsed again to ensure that any residual hair fragments or particles of Dermabond were removed. This resulted in an STSG of the scalp, devoid of any embedded hair and mechanical damage (Figure.3). The depilated grafts were then grafted to the recipient site.

## Discussion

The scalp is a hair-bearing area, and STSGs from the scalp contain a large amount of residual hair particles after harvesting. Residual hair within the graft has been associated with foreign body reaction similar to pseudofolliculitis [2-4]. The hair in the graft can lead to persistent inflammation, which can initiate infection [5]. There is a need to remove the hair and hair particles to limit this foreign body reaction and avoid grafting hair-bearing tissue to a non-hair bearing area of the body allowing for improved immediate aesthetics of the recipient site.

Many techniques have been used intraoperatively to remove hair from grafts prior to graft application. These techniques include the use of forceps to remove the hair and saline agitation. The most recent technique described in the English language literature involves the use of Ioban and saline to remove hair from scalp STSGs [5]. This technique involves applying an Ioban adhesive to the epidermal and dermal aspect of the graft and then removing the Ioban, the adhesive attaches to the hair and pull it out of the graft as it is peeled off the graft. The graft is then washed in saline. Although this technique has many merits, it cannot be used in patients who are allergic to Ioban. In addition, the process requires multiple rounds of Ioban application to a single graft which may subject the graft to mechanical damage.

In this article, we present a technique for intraoperative hair removal from the scalp donor STSG. This method uses Dermabond and saline washing for graft hair removal. Dermabond is a topical skin adhesive containing 2-octylcyanoacrylate [6]. To the best of our knowledge, this is the first report of this technique in the English language literature. This technique produces a virtually hair-free STSG with very little manipulation of the graft, when done correctly. Additionally, this technique can be used in patients with allergies to iodine. The potential downside of our technique is the cost associated with the use of Dermabond. 

## Conclusions

Intraoperative Dermabond depilation of STSGs is safe, easy, effective, and expeditious.
